# Supplementary material for: Transcriptional Regulation of zma-MIR528a by Action of Nitrate and Auxin in Maize
Source: Int J Mol Sci. 2022 Dec 11;23(24):15718. doi: 10.3390/ijms232415718 (PMC9779399; doi:10.3390/ijms232415718)
Supplement: Supplementary file 1 [file ijms-23-15718-s001.zip › Lujan-Soto_etal_2022_Supplementary_Figures_IJMS.pdf]

Supplementary Material

1 Supplementary Figures

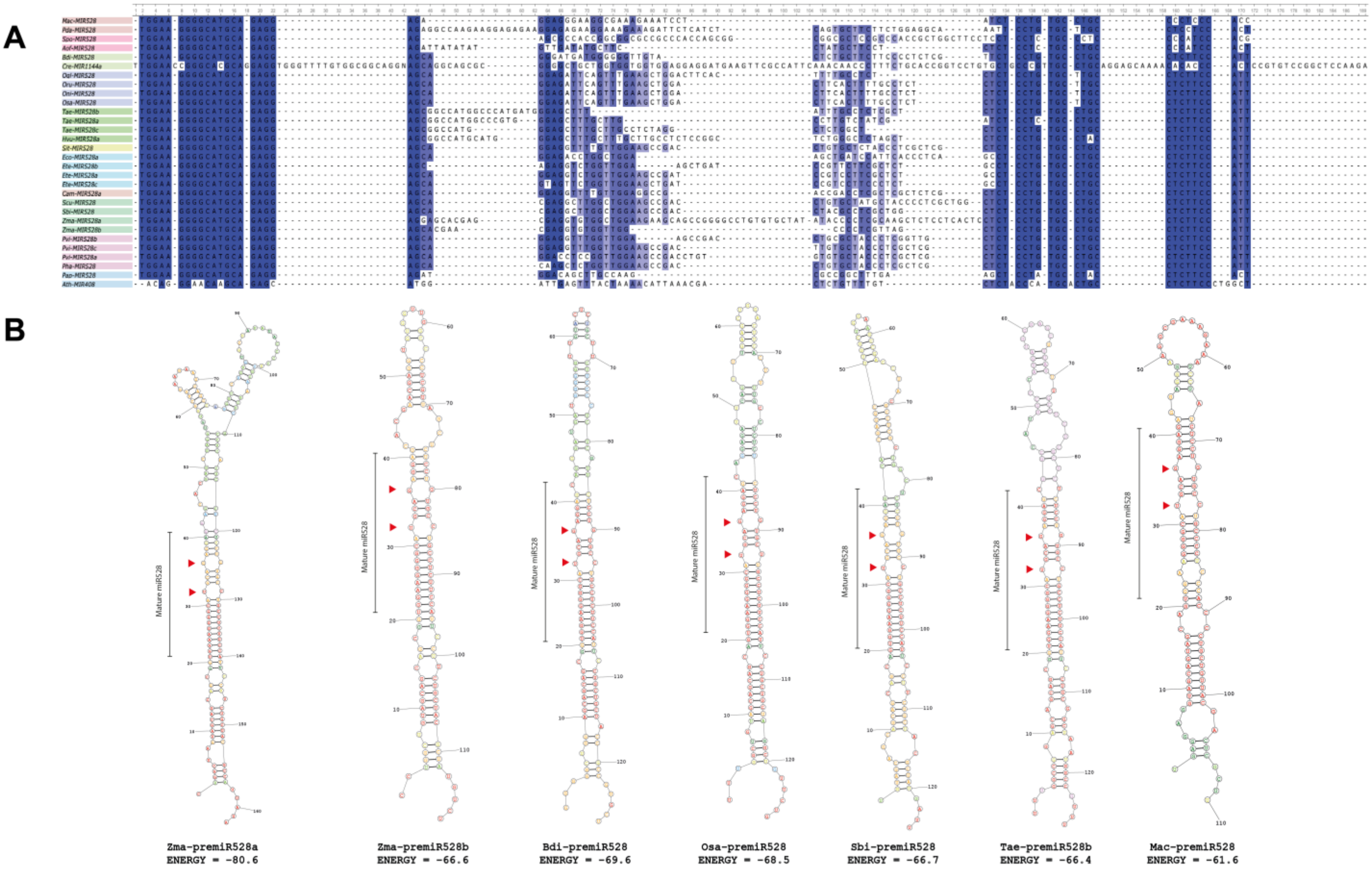

Figure S1.- Sequence and structure conservation between miR528 precursors.

(A) Sequence alignment of miR528 precursors in different monocots. Blue highlighted nucleotides represent conserved regions. The lighter blue indicates lesser conservation. (B) Predicted secondary structures of miR528 precursors for several monocots obtained with the RNAFold program. At the bottom, minimum free energy of each structure is displayed. Each structure is colored based on the base-pairing probabilities derived from the partition function. As a nucleotide is more red-colored, higher is the likelihood of the predicted structure. Positions with conserved mismatches in the duplex region (12th and 16th nt of the mature sequence) across precursors are indicated with red triangles. The portion of each structure corresponding to the mature miRNA is indicated with a black line.

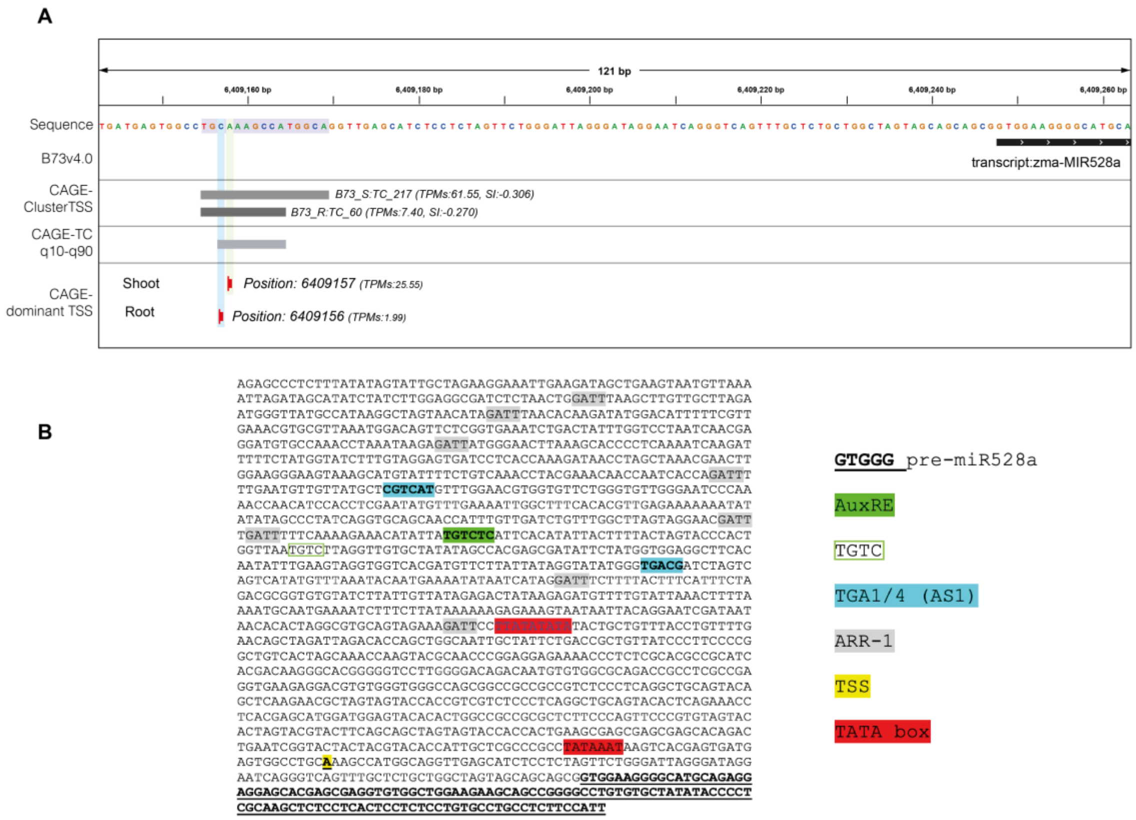

Figure S2.- CAGE TSS data analysis and the promoter sequence of zma-*MIR528a*. (A) Representation of public CAGE-Seq (Cap Analysis Gene Expression and deep Sequencing) data [GSE70251] of B73 shoot and root tissues for the genomic localization of zma-*MIR528a* TSS. CAGE tags were grouped into CAGE cluster-TSS (horizontal grey lines) with the dominant TSS position indicated with red blocks. TPM: tags per million. SI: shape index. (B) The analyzed promoter sequence (1500 bp) of zma-*MIR528a*. The underlined portion corresponds to annotated pre-miR528a, and the colored regions represent selected regulatory elements present in the promoter: Auxin Response Element (AuxRE, green), binding motifs for TGA1/4 proteins known as ACTIVATION SITE 1 (TGA1/4; AS-1, blue), ARR-1 binding elements from Arabidopsis (ARR-1, grey), and TATA boxes (red). The dominant TSS position is highlighted in yellow, bold, and underlined.

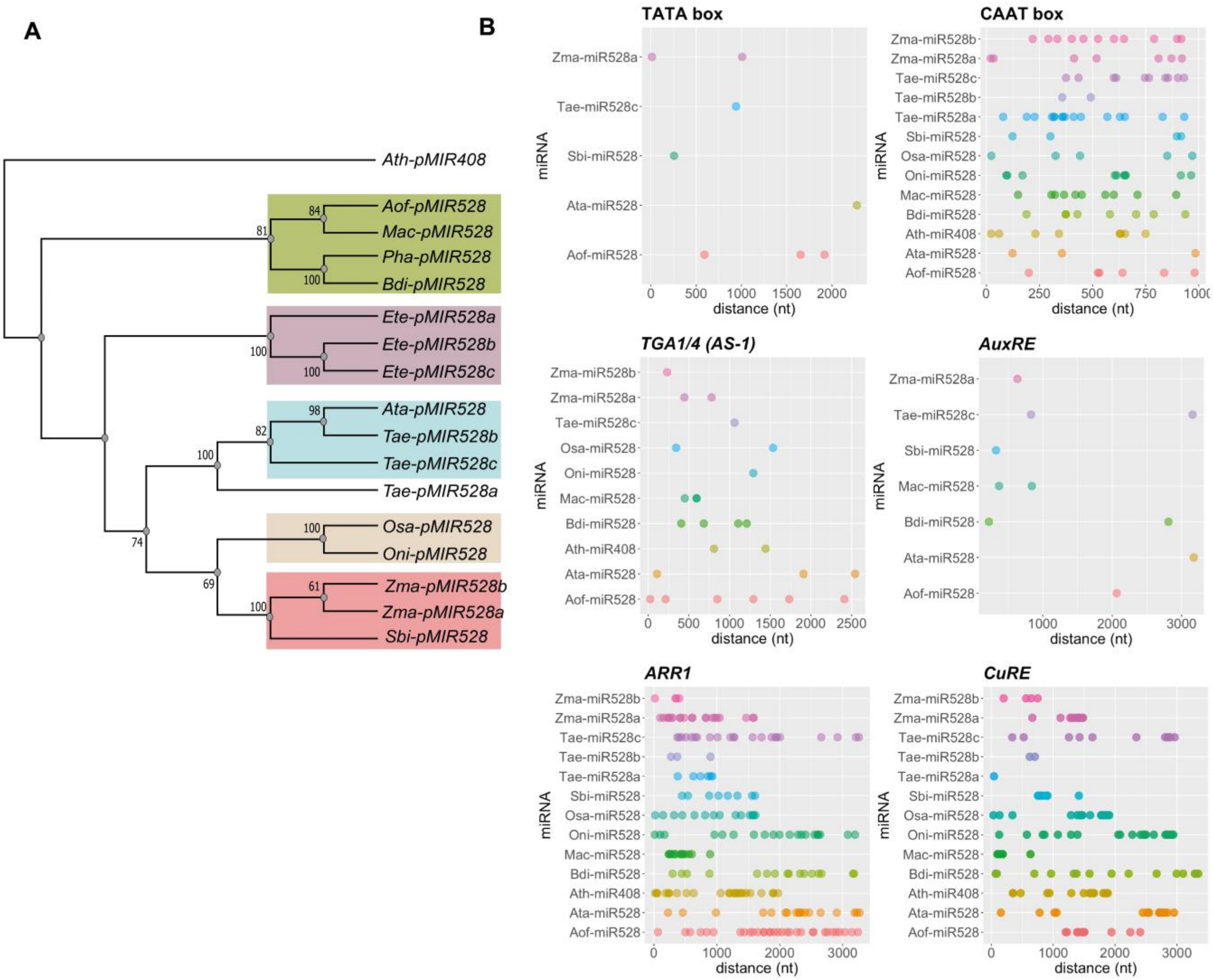

Figure S3.- Conserved *cis* regulatory elements present in *MIR528* promoters in monocots. (A) Phylogenetic analysis of sixteen *MIR528* promoters in monocots. An unrooted tree was constructed by aligning the upstream regions (≈2000 nt from the precursor mapping site) for promoter homology comparisons. Sequences were aligned using nucleotide optimized MUSCLE algorithm with 50 iteration cycles. The phylogenetic tree was constructed with MEGA X, using the Maximum Likelihood (ML) method and the best-adjusted model (*3-parameter Tamura*) with 1000 bootstrap replications. Numbers at the nodes indicate the bootstrap values. (B) Schematic representation of predicted transcription factor binding sites (TFBS) for *MIR528* genes in several monocots. Location of TFBS upstream of pre-miR528 is shown from left to right on the horizontal axis. Dots represent the relative position of each TFBS in different species (denoted by color).

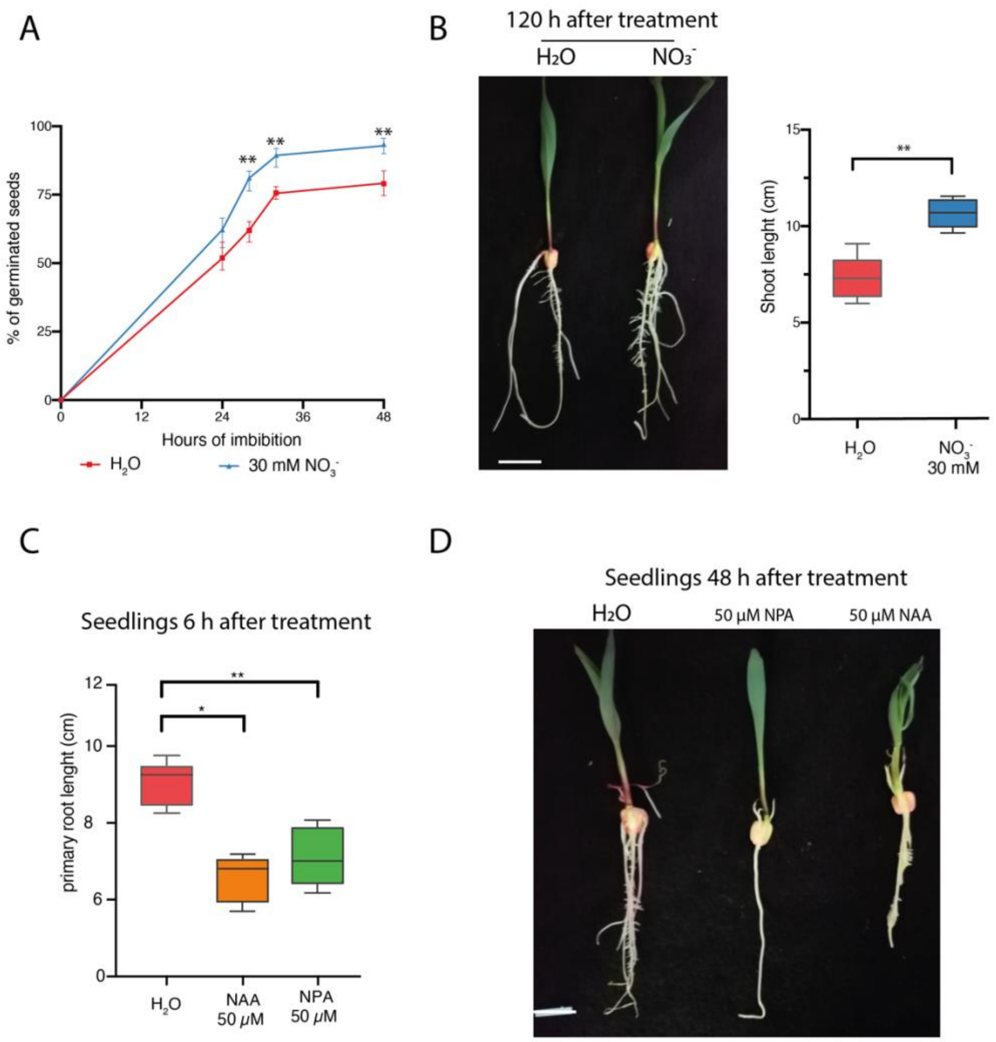

Figure S4.- Physiological effects of the application of nitrate or auxin treatment during maize seed germination and seedling establishment. (A) Percentage of germinated maize seeds imbibed in H_2O (control, red line) or 30 mM KNO_3 (blue line) for up to 48 h. Bars indicate standard deviation (n=3 independent biological replicates, ** $p < 0.01$, Student's t-test comparing treated samples to control for each time-point). (B) Effect of extended incubation time under high nitrate conditions. Seedlings were incubated for 120 h either under control (H_2O) or high nitrate (30 mM KNO_3, NO_3^-) conditions and the shoot length was evaluated (left panel). Error bars indicate standard deviation (n=3 independent biological replicates, ** P <0.01 from Student's t-test comparing treatments). White bar on images corresponds to 1 cm. (C) Quantification of the primary root length of maize seedlings 6 h after auxin (NAA) application on NPA-treated seeds as compared with H_2O or NPA alone controls. Error bars indicate standard deviation from 3 independent biological replicates (* P <0.05 , ** P <0.01; data were analyzed by a one-way ANOVA with Tukey post hoc test for multiple comparisons). (D) Effect of extended treatment (48 h) with 50 µM NAA of NPA-treated seedlings, as compared to those imbibed in H_2O or 50 µM NPA alone. Scale bar = 1 cm.

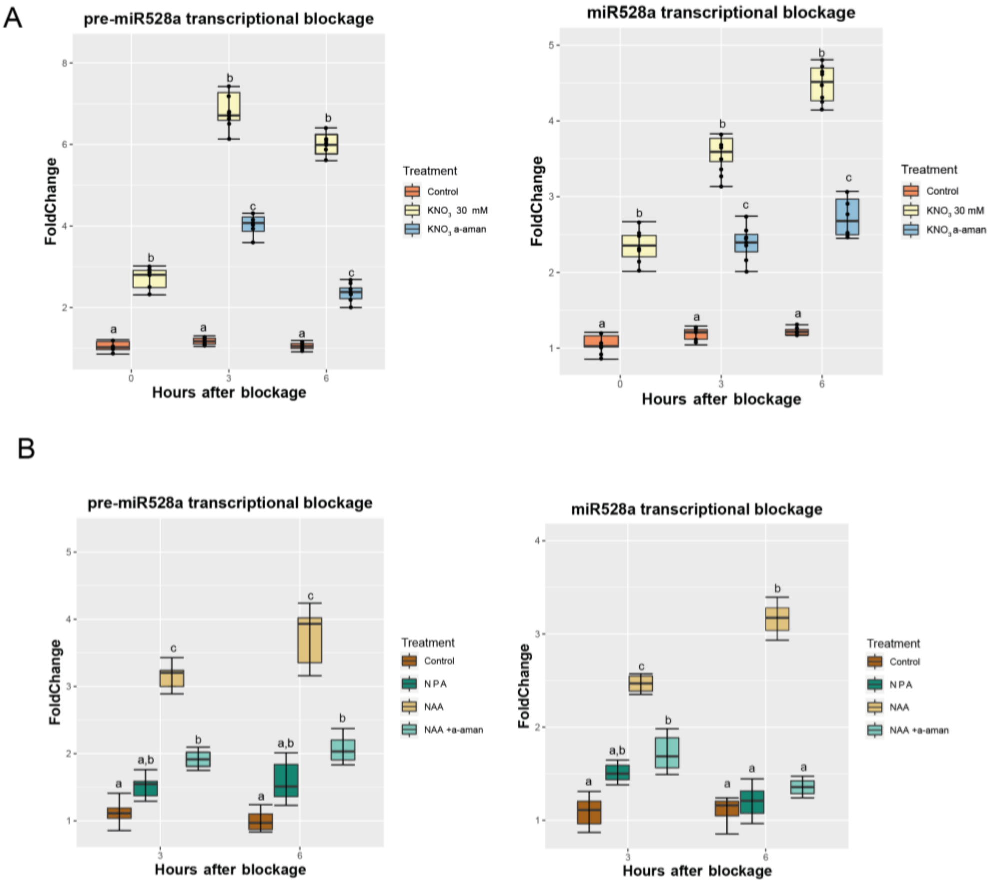

Figure S5.- The up-regulation of pre-miR528a and mature miR528 levels by nitrate and auxin is significantly reduced by inhibition of RNA pol II activity. Relative expression levels of precursor (left panel) and mature (right panel) miR528 after treatment with 30 mM KNO_3 (A) or 50 µM NAA (B) in the presence of α-amanitin (25 µM). For nitrate stimulus, the inhibition of RNA pol II activity was performed for seeds previously incubated with H_2O (control) or 30 mM KNO_3 solution for about 42 h, corresponding to “0 h” time-point. After this, nitrate-treated seeds were selected and incubated with the mixture of KNO_3 and α-amanitin with sampling at 3 and 6 h hours after blockage. On the other hand, exogenous auxin and α-amanitin were added simultaneously to seeds previously incubated with NPA, and samples were taken from each group after 3 and 6 h of blockage. Data were normalized using 18S rRNA (for precursor) and U6 snRNA (for mature miRNA) expression. Boxes that do not share at least a letter differ significantly ($p < 0.05$) from control sample at each time-point. Bars represent standard errors of means from at least three independent replicate experiments. Statistically significant difference between samples with treatments and samples with treatments together with a-Amanitin is denoted with different letters ($p \leq 0.05$, one-way ANOVA with Tukey post hoc test comparing treated samples to control for each time point).

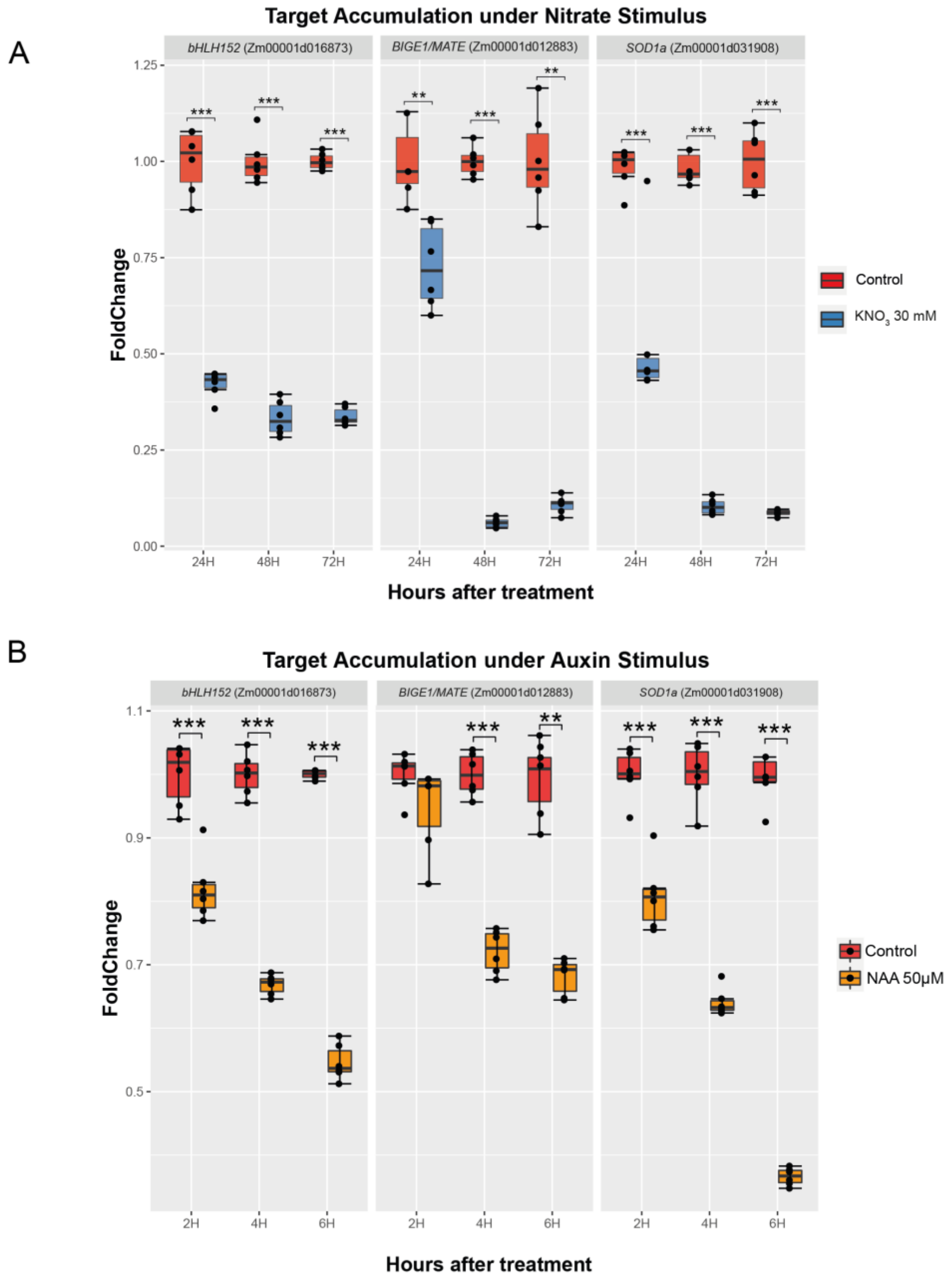

Figure S6.- Negative impact of nitrate and auxin treatments on miR528 target accumulation. The relative accumulation levels of *bHLH152 (Zm00001d016873;* right*), MATE/BIGE1 (Zm00001d012883*, middle*)*, and *SOD1a* (Zm00001d031908; left) miR528 targets in samples treated with 30 mM KNO_3 (A) or 50 µM NAA (B). Data were normalized using 18S rRNA expression. Error bars represent standard errors of means from at least three independent replicate experiments. Data were analyzed by one-way ANOVA with Tukey post hoc test comparing treated samples to control for each time point (** *P* >0.01, and *** *P*< 0.001)

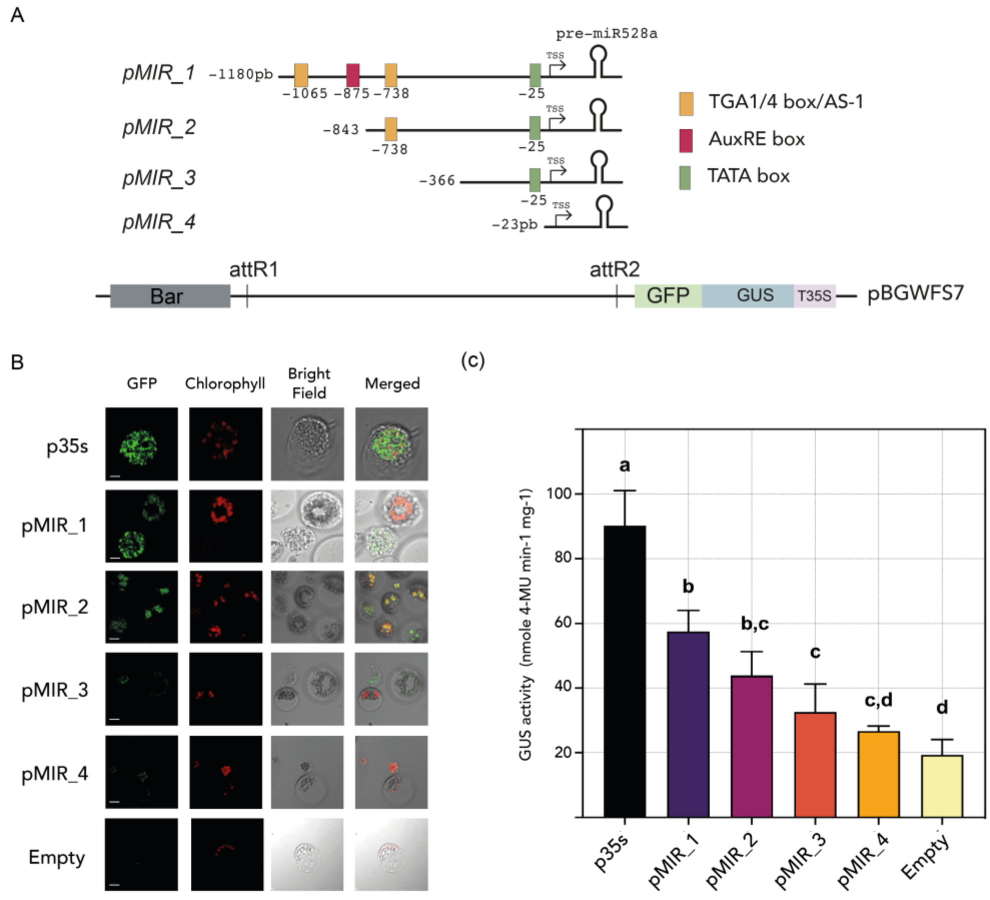

Figure S7.-Zma-*MIR528a* promoter drives basal reporter expression in maize protoplasts. (A) Full-length zma-*MIR528a* promoter (pMIR_1) and 5’ deletion fragments (pMIR_2-4) were cloned into the pBGWFS7.0 vector upstream the eGFP/GUS reporters (B) Transient expression of eGFP controlled by each zma-*MIR528a* promoter version (pMIR_1-4) under control conditions. Scale bar=20 μm (C) Fluorometric GUS assay of maize protoplasts transfected with pMIR_1-4 constructs and empty (negative control) or 35S:GUS (positive control) vectors under control conditions. Columns represent the mean of three independent replicates with three technical replicates each. Data were analyzed by a one-way ANOVA test with multiple comparisons by the Tukey post hoc test. Bars that do not share at least a letter differ significantly ($p < 0.05$) from each other.

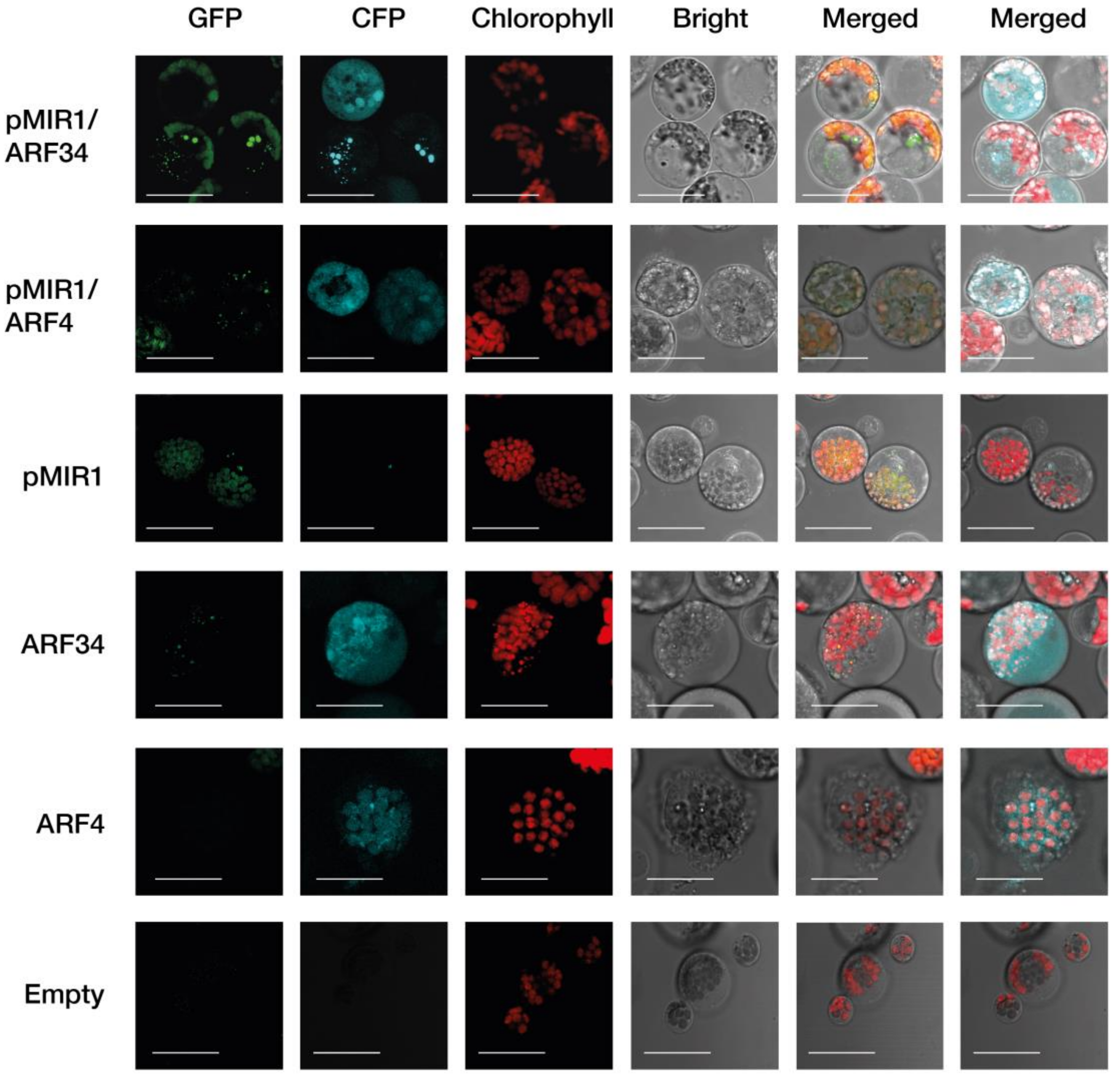

Figure S8. Protoplast co-transfection assays. Maize mesophyll protoplasts were transiently co-transfected with a reporter construct (pMIR_1::GFP/GUS) and effector plasmid (35SCaMV::ZmARF34-CFP or 35SCaMV::ZmARF4-CFP). GFP and CFP were analyzed by confocal microscopy and each signal was merged with the bright field image. Transient transformation with pMIR_1 alone was used to set basal expression for the reporter construct. Scale bar = 40 µm.

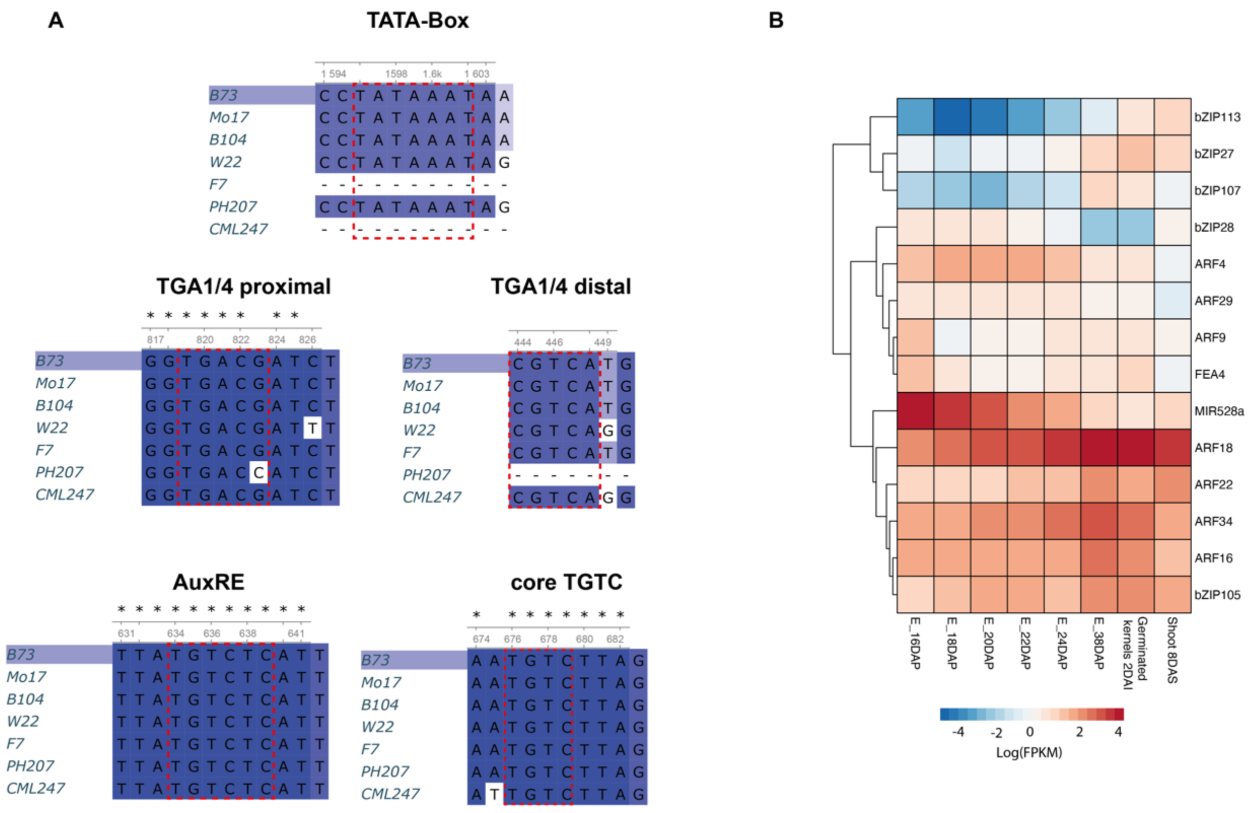

Figure S9. The TATA-box, TGA1/4, and AuxRE sites of the Zma-*MIR528a* promoter are conserved among lines. (A) *MIR528a* promoter sequences of representative inbred maize lines were aligned against the B73 sequence (reference) to explore the conservation of each TFBS analyzed in our study. The red dotted line surrounds the core sequence for each motif. (B) Expression data of several TFs that potentially recognize TGA1/4 and AuxRE sites in the promoter of zma-*MIR528a*. Read counts (as log FPKM) of maize homologs of TGA1/4 from *A. thaliana* (FEA4, bZIP105,bZIP107, bZIP113, bZIP27, bZIP28) and some ARFs (ARF34, ARF4, ARF29, ARF22, ARF16, ARF18, ARF9) from tissues similar to the ones used in our work (immature embryo at 16,18,20,22 24, 38 days after pollination; germinating kernels 2 days after imbibition (2DAI), and shoot leaf 8 days after sowing, 8DAS) were analyzed.
